# An efficient weighted tag SNP-set analytical method in genome-wide association studies

**DOI:** 10.1186/s12863-015-0182-3

**Published:** 2015-03-13

**Authors:** Bin Yan, Shudong Wang, Huaqian Jia, Xing Liu, Xinzeng Wang

**Affiliations:** College of Mathematics and Systems Science, Shandong University of Science and Technology, Qingdao, Shandong 266590 China; College of Computer and Communication Engineering, China University of Petroleum, Qingdao, Shandong 266580 China; State Key Laboratory of Mining Disaster Prevention and Control Co-founded by Shandong Province and the Ministry of Science and Technology, Shandong University of Science and Technology, Qingdao, Shandong 266590 China

**Keywords:** Association test, GWAS, Linkage disequilibrium, SNP-set, Tag SNP

## Abstract

**Background:**

Single-nucleotide polymorphism (SNP)-set analysis in Genome-wide association studies (GWAS) has emerged as a research hotspot for identifying genetic variants associated with disease susceptibility. But most existing methods of SNP-set analysis are affected by the quality of SNP-set, and poor quality of SNP-set can lead to low power in GWAS.

**Results:**

In this research, we propose an efficient weighted tag-SNP-set analytical method to detect the disease associations. In our method, we first design a fast algorithm to select a subset of SNPs (called tag SNP-set) from a given original SNP-set based on the linkage disequilibrium (LD) between SNPs, then assign a proper weight to each of the selected tag SNP respectively and test the joint effect of these weighted tag SNPs. The intensive simulation results show that the power of weighted tag SNP-set-based test is much higher than that of weighted original SNP-set-based test and that of un-weighted tag SNP-set-based test. We also compare the powers of the weighted tag SNP-set-based test based on four types of tag SNP-sets. The simulation results indicate the method of selecting tag SNP-set impacts the power greatly and the power of our proposed method is the highest.

**Conclusions:**

From the analysis of simulated replicated data sets, we came to a conclusion that weighted tag SNP-set-based test is a powerful SNP-set test in GWAS. We also designed a faster algorithm of selecting tag SNPs which include most of information of original SNP-set, and a better weighted function which can describe the status of each tag SNP in GWAS.

## Background

With the development of high throughput genotyping technology, more and more biologists use GWAS to analyze the associations between disease susceptibility and genetic variants [1-3]. Although standard analysis of a case–control GWAS has identified many SNPs and genes associated with disease susceptibility [4-6], it suffers from difficulties in detecting epistatic effects and reaching the significant level of Genome-wide [7,8]. As an alternative analytical strategy, some researchers put forward association analytical approaches based on SNP-set [8-14], which have obvious advantages over those based on individual SNP in improving test power and reducing the number of multiple comparisons.

Max-single is the simplest method using the maximum *χ*^2^ statistic of all SNPs to compute the p-value of the SNP-set [9]. However, this method might not be optimal as it does not utilize the LD structure among all genotyped SNPs, especially when the disease locus has more than one in SNP-set. Fan and Knapp [10] used a numerical dosage scheme to score each marker genotype and compared the mean genotype score vectors between the cases and controls by Hotelling’s *T*^2^ statistic. Compared with the former, the later makes full use of the LD information, but the degree of freedom of Hotelling’s *T*^2^ increases greatly. Mukhopadhyay [11] constructed kernel-based association test (KBAT) statistic, which compared the similarity scores within groups (case and control) and between groups. The simulation results indicated that KBAT has stronger power than multivariate distance matrix regression (MDMR) by Wessel [12] and Z-global by Schaid [9]. The principal component analysis (PCA) was first applied to analyze the association between disease susceptibility and SNPs by Gauderman [14]. He extracted linearly independent principal components (PCs) from the expression vectors of all SNPs in SNP-set and tested the association between qualitative trait and PCs under logistic model. Compared with the above method, PCA gets more favour for the improved power because great reduction of the degree of freedom remedies the limitation of the information loss. Lately, Wu [8] proposed sequence kernel association test (SKAT) based on logistic kernel-machine model, which allows complex relationships between the dependent and independent variables [15]. The simulation results showed that SKAT gains higher power than individual-SNP analysis.

All the above methods are involved the selection of SNP-sets and the quality of SNP-set can further affect the test power greatly. As an alternative solution, we propose selecting some representative SNPs (called tag SNP-set) from the original SNP-set [16-18] and then designing a proper weighted function on the association test to remedy the information loss in the process of forming tag SNP-set. The existing algorithms of selecting tag SNPs, such as pattern recognition methods proposed by Zhang [16] or Ke [17], statistical method put forward by Stram [18] and software tagsnpsv2 [19] written by Stram, are with high time complexity. Therefore, we first propose a novel fast algorithm of selecting tag SNPs based on the LD structure among the genotyped SNPs. Then design a weighted function in constructing tag SNP-set-based test (called weighted tag SNP-set-based test). The intensive simulation results indicate that our method has much higher power than those of tests based on original SNP-set, tag SNP-set and weighted original SNP-set.

The remainder of this paper is organized as follows. In the next section, we will introduce the proposed fast algorithm of selecting tag SNP-set, weighted function, and statistics KBAT and SKAT used in this paper. Then we will list simulation scenarios and simulation results of the comparison of the weighted tag SNP-set-based test and the weighted original SNP-set-based test. The analysis and discussion of the results are shown at the end of this paper.

## Methods

### Notations

Assumed that there are *p* SNP loci to be tested in the original SNP-set, and *n* independent subjects in a case–control GWAS. Select randomly *m* subjects *i*_1_, *i*_2_, ⋯, *i*_*m*_ from the *n* subjects, *i*_*j*_ ∈ {1, 2, ⋯, *n*}, *j* = 1, 2, ⋯, *m*, *m* ≪ *n*. We intend to test the haplotypes at all the *p* SNP loci of the *m* subjects. Thus we get 2 *m* haplotypes, where every allele at each locus only has two possibilities 0 or 1, representing the major allele and the minor allele respectively. Let *Z*_*i*_ = (*z*_*i*1_, *z*_*i*2_, …, *z*_*ip*_) denote all the alleles of the *i*^*th*^ haplotype at all the *p* SNP loci (*i* = 1, 2, ⋯, 2 *m*), where *z*_*ij*_ ∈ {0, 1}, *i* = 1, 2, ⋯, 2 *m*, *j* = 1, 2, ⋯, *p*. For the remaining *n-m* subjects ${i}_1^{\hbox{'}},{i}_2^{\hbox{'}},\cdots, {i}_{n-m}^{\hbox{'}},{i}_j^{\hbox{'}}\in \left\{1,2,\cdots, n\right\},j=1,2,\cdots, n-m,$ we only need to consider the genotypes of their *s* tag SNP loci *l*_1_, *l*_2_, ⋯, *l*_*s*_, *s* ≪ *p*. Obviously, this reduces greatly the cost of genotyping. Let ${G}_k=\left({g}_{k{l}_1},{g}_{k{l}_2},\dots, {g}_{k{l}_s}\right)$ denote the genotype value vector of the *k*^*th*^ subject at all the *s* tag SNP loci (*k* = 1, 2, ⋯, *n*), where the genotype value *g*_*kj*_ = 0, 1, 2. corresponds to homozygotes for the major allele, heterozygotes and the homozygotes for minor allele under the additive model, respectively (*k* = 1, 2, ⋯, *n*, *j* = *l*_1_, *l*_2_, ⋯, *l*_*s*_). Let *y*_*i*_ denote the qualitative trait of the *i*^*th*^ subject and *y*_*i*_ = 1 for case, *y*_*i*_ = 0 for control, *i* = 1, 2, ⋯, *n*.

### Fast algorithm of selecting tag SNPs

Up to now, many approaches of grouping the original SNP-sets have been proposed, such as gene-, LD structure-, biological pathway- and complex network clustering-based approaches [8]. In our study, we employ the gene-based approach, namely treat all the SNPs in a gene as an original SNP-set. We select a subset of SNPs from the original SNP-set, in which each SNP is the representative with high expression correlation. Obviously, the subset includes most of information of the original SNP-set and we define it as the tag SNP-set of the original SNP-set, tag SNP-set for short without confusion. We divide the original SNP-set into some subsets by the rules that the SNPs in the same subset have high expression correlations among individuals and the SNPs in different subsets have low correlations, then choose one SNP of each subset (regarded as a tag SNP) as the representative of this subset. All the tag SNPs forms a tag SNP-set. The detailed algorithm is as follows.

Input haplotypes *z*_*ij*_ of all the *p* loci of the *m* subjects, *i* = 1, 2, ⋯, 2 *m*, *j* = 1, 2, ⋯, *p*.

Step 1 compute the coefficient *R*_*ij*_ of LD describing the correlation between SNP *i* and SNP *j* [20],

$$ {R}_{ij}={R}_{ji}={\left\{\frac{1}{\left(2m-1\right){S}_i{S}_j}{\displaystyle \sum_{k=1}^{2m}}\left({z}_{ki}-{\overline{z}}_i\right)\left({z}_{kj}-{\overline{z}}_j\right)\right\}}^2,i,j=1,2,\cdots, p,i\ge j, $$

where ${\overline{z}}_i$ and *S*_*i*_ denote the mean and the variance of *z*_·*i*_ respectively. *t* is a threshold in the interval [0, 1]. We set *t* = 0.9 based on a series of experiments. If *R*_*ij*_ > *t* or *i = j*, let *N*_*ij*_ = 1, otherwise *N*_*ij*_ = 0, *i*, *j* = 1, 2, ⋯, *p*, *i* ≥ *j*. Let *S* = ∅, *B* = {1, 2, …, *p*}.

Step 2 choose an element *k* from *B* randomly. Let

$$ Q=\left\{k\right\},k\in B,B=B-\left\{k\right\}. $$

Step 3 if there exists *N*_*mn*_ = 1, *m* ∈ *Q*, *n* ∈ *B*, then let *Q* = *Q* + {*n*}, *B* = *B* − {*n*}, and go to Step 3; Otherwise go to Step 4.

Step 4 determine the tag SNP of the subset *Q* grouped in Step 3. Namely, let

$$ {t}_Q= min\left\{i\left|\underset{i\in Q}{max}{R}_i={\displaystyle \sum_{j\in Q}}{R}_{ij}\right.\right\},\kern0.24em S=S+\left\{{t}_Q\right\}. $$

Step 5 if *B ≠ ∅*, go to Step 2; Otherwise Stop.

Output tag SNP-set *S*

We compare the time complexity of the above algorithm and software tagsnpsv2 [19], listed in Table 1. Table 1 shows that our algorithm of selecting tag SNPs has absolute advantage over software tagsnpsv2 from the view of time complexity.

**Table 1:** **The comparisons of time complexity between our algorithm and tagsnpsv2**

**Method**	**Running time** ^**1**^ **(about 10 from 163)**	**Running time** ^**1**^ **(about 36 from 163)**
Our algorithm	Less than 1 minute	Less than 1 minute
tagsnpsv2	About 35 minutes	About 55 minutes

^1^Its execution is on the ENr321 gene and a server (Intel(R) Core(TM) i3-3240 T CPU @2.90GHz2.90GHz, 4GB Windows 8).

### Weighted function

Among the analytical methods based on SNP-set, weighted analysis tends to increase the power [8]. The square of *χ*^2^ statistic of single SNP is used to weight the corresponding SNP in our research. The detailed formula [21] of computing the weight *w*_*i*_ corresponding to the *i*^*th*^ SNP is

$$ {w}_i={\left\{\frac{{\left( ad-bc\right)}^2\left(a+b+c+d\right)}{\left(a+b\right)\left(a+c\right)\left(c+d\right)\left(b+d\right)}\right\}}^2, $$

where *a, b, c, d* are the observed data of *i*^*th*^ SNP in case and control.

### Kernel-based association test (KBAT)

Mukhopadhyay [11] proposed KBAT statistic based on U-statistic [22]. Let $$ {\overline{U}}_l^k={\displaystyle {\sum}_{i<j}{h}_l^k}\left({g}_i^k,{g}_j^k\right)/{m}_l $$ denote U-statistic of the *k*^*th*^ SNP in the *l*^*th*^ group, where *l = 1, 2* represent case and control respectively; $$ {m}_l={C}_{n_l}^2, $$*n*_*l*_ is the number of subjects in the *l*^*th*^ group; the $$ {h}_l^k\left(\cdot, \cdot \right) $$ is the kernel, allele match kernel (AM) function [11] is used in our study. Let $$ {W}_k={\displaystyle {\sum}_{l=1}^2{\sum}_{i<j}}{\left[{h}_l^k\left({g}_i^k,{g}_j^k\right)-{\overline{U}}_l^k\right]}^2 $$ and $$ {B}_k={\displaystyle {\sum}_{l=1}^2{m}_{\mathrm{l}}}\left({\overline{U}}_l^k-{\overline{U}}_k\right) $$ represent the quadratic sum of the kernel score of *k*^*th*^ SNP within group and between groups, respectively, where $$ {\overline{U}}_k=\left({\overline{U}}_1^k+{\overline{U}}_2^k\right)/2. $$ Mukhopadhyay employed KBAT statistic to test the association between SNP-set and phenotype. The statistic is

$$ KBAT=\frac{{\displaystyle {\sum}_{k=1}^p}{B}_k}{{\displaystyle {\sum}_{k=1}^p}{W}_k}. $$

Although KBAT statistic is constructed using *F* distribution, it does not obey *F* distribution [11]. We compute the p-value by a permutation procedure under the null model to count the empirical quantiles of KBAT statistic. The details of KBAT method can be found in [11].

In our research, we perform original SNP-set-based test and tag SNP-set-based test using KBAT. For convenience to describe, we denote the original SNP-set-based test as KBAT, and tag SNP-set-based test as KBAT-tag. In weighted analysis, we compare the powers of the tests based on weighted KBAT with weighted KBAT-tag.

### Sequence kernel association test (SKAT)

To further verify the effectiveness of our method, we also conduct the similar comparisons using sequence kernel association test (SKAT) statistic instead of KBAT. For the *i*^*th*^ subject, we use the following model (1) to describe the correlation between the phenotype and the genotypes:

1 $$ \mathrm{logit}P\left({y}_i=1\right)={\alpha}_0+{\alpha}_1{x}_{i1}+\cdots +{\alpha}_m{x}_{im}+h\left({z}_{i1},{z}_{i2},\cdots, {z}_{ip}\right) $$

where *α*_0_ is an intercept term, *α*_1_, ⋯, *α*_*m*_ are regression coefficients and *x*_1_, ⋯, *x*_*m*_ are the environmental and demographic covariates. The correlation is completely defined by function *h*(⋅) and $h\left({Z}_i\right)={\displaystyle {\sum}_{j=1}^n{\gamma}_j}K\left({Z}_i,{Z}_j\right)$ according to Representer Theorem [23], where *γ*_1_, ⋯, *γ*_*n*_ are the coefficients. The mean and variance of *h*(*z*) are 0 and *τK* respectively offered by Liu [24]. We can consider the null hypothesis *h*(*z*) = 0 by testing *τ* = 0, and Wu [8] proposed to test *τ* = 0 using the score statistic *Q* introduced by Zhang and Lin [25]. The *Q*-statistic is

$$ Q=\frac{{\left(y-{\widehat{p}}_0\right)}^{\hbox{'}}K\left(y-{\widehat{p}}_0\right)}{2}, $$

where $\mathrm{logit}\kern0.5em {\widehat{p}}_{0_i}={\widehat{\alpha}}_0+{\widehat{\alpha}}_1{x}_{i1}+\cdots +{\widehat{\alpha}}_m{x}_{im},$*Q* obeys *χ*^2^ distribution with scale parameter *κ* and degree of freedom *v*. The details of SKAT method can be found in [8]. We also use the notations SKAT, SKAT-tag similar to KBAT.

### Simulations

To evaluate the performance of weighted tag SNP-set analytical method, we conduct extensive simulations. All causal SNPs used in our study are assumed to increase the disease risk, because KBAT are not affected by the direction of effect [11].

*HTR2A*, associated with Schizophrenia and Obsessive-compulsive disorder [26,27], is a 62.66-kb-long gene with 169 HapMap [28] SNPs and is located at 13q14-q21. A total of 34 out of 169 SNPs genotyped by Illumina Human Hap 650v3 array [29] are used to be the causal SNPs in simulations. We consider *HTR2A* gene for instance and use the HAPGEN2 [30] to generate SNP data at each locus on the basis of the LD structure of the CEU samples of the International HapMap Project.

To verify the effectiveness of our proposed method, we first generate replicated datasets at the 169 SNP loci on the *HTR2A* gene in nine different scenarios using HAPGEN2, where each data set includes 500 cases and 500 controls. Then choose one from the replicated data sets for each scenario and 200 haplotypes of 50 cases and 50 controls from this set randomly as the considered haplotypes used to form the tag SNP-set by the algorithm of selecting tag SNPs mentioned in the methods. In the first scenario, 5000 replicated data sets are generated under the null disease model and 1000 replicated data sets are generated under different disease models which assume the same heterozygote disease risk 1.25 and same homozygote disease risk 1.5 for other scenarios. We assume there is only one causal SNP in scenario 2 and two causal SNPs specified randomly in scenarios 3–9. Both of the two causal SNPs are genotyped by Illumina Human Hap 650v3 array in scenario 3–5, only one is genotyped in scenarios 6–8, and no causal SNPs are genotyped in scenarios 9. The minor allele frequency (MAF), the mean *R*^2^ with genotyped SNPs and the distance between the causal SNPs are also different. The detailed parameters for scenarios 2–9 are listed in Table 2.

**Table 2:** **Simulation parameters in scenarios 2-9**

**Scenario**	**No. of causal SNP**	**Causal SNP**	**The position of causal SNP**	**Genotyped**	**MAF** ^**1**^	**Mean** ***R*** ^**2**^ **with the genotyped SNPs** ^**2**^
2	1	Each of all the 34 SNPs				
3	2	rs977003	46313002	Yes	0.449	0.0853
		rs9534511	46366581	Yes	0.442	0.178
4	2	rs3803189	46306571	Yes	0.107	0.0474
		rs977003	46313002	Yes	0.449	0.0853
5	2	rs3803189	46306571	Yes	0.107	0.0474
		rs731779	46350039	Yes	0.161	0.2478
6	2	rs9526246	46347862	No	0.462	0.2164
		rs9534511	46366581	Yes	0.442	0.178
7	2	rs3803189	46306571	Yes	0.107	0.0474
		rs9526246	46347862	No	0.462	0.2164
8	2	rs3803189	46306571	Yes	0.107	0.0474
		rs3742278	46317578	No	0.158	0.0535
9	2	rs6561333	46318313	No	0.466	0.1127
		rs9526246	46347862	No	0.462	0.2164

^1^minor allele frequency.

^2^the average of *R* ^2^ between the causal SNP and 34 genotyped SNPs.

## Results

### The preliminary validation using KBAT Type I error rate evaluation

We simulate 5000 replicated data sets to estimate type I error rate in scenario 1. The detailed results are listed in Table 3 at the significance level of 0.005, 0.01 and 0.001 respectively. Table 3 indicates that the type I error of our method can be controlled.

**Table 3:** **Type I error rate in scenario 1 for KBAT**

**Significance level**	**KBAT**	**KBAT-tag**	**Weighted KBAT**	**Weighted KBAT-tag**
0.05	0.049	0.05	0.048	0.046
0.01	0.0096	0.0096	0.0098	0.0092
0.001	0.0008	0.0012	0.0008	0.001

### Power evaluation

**Figure 1:**
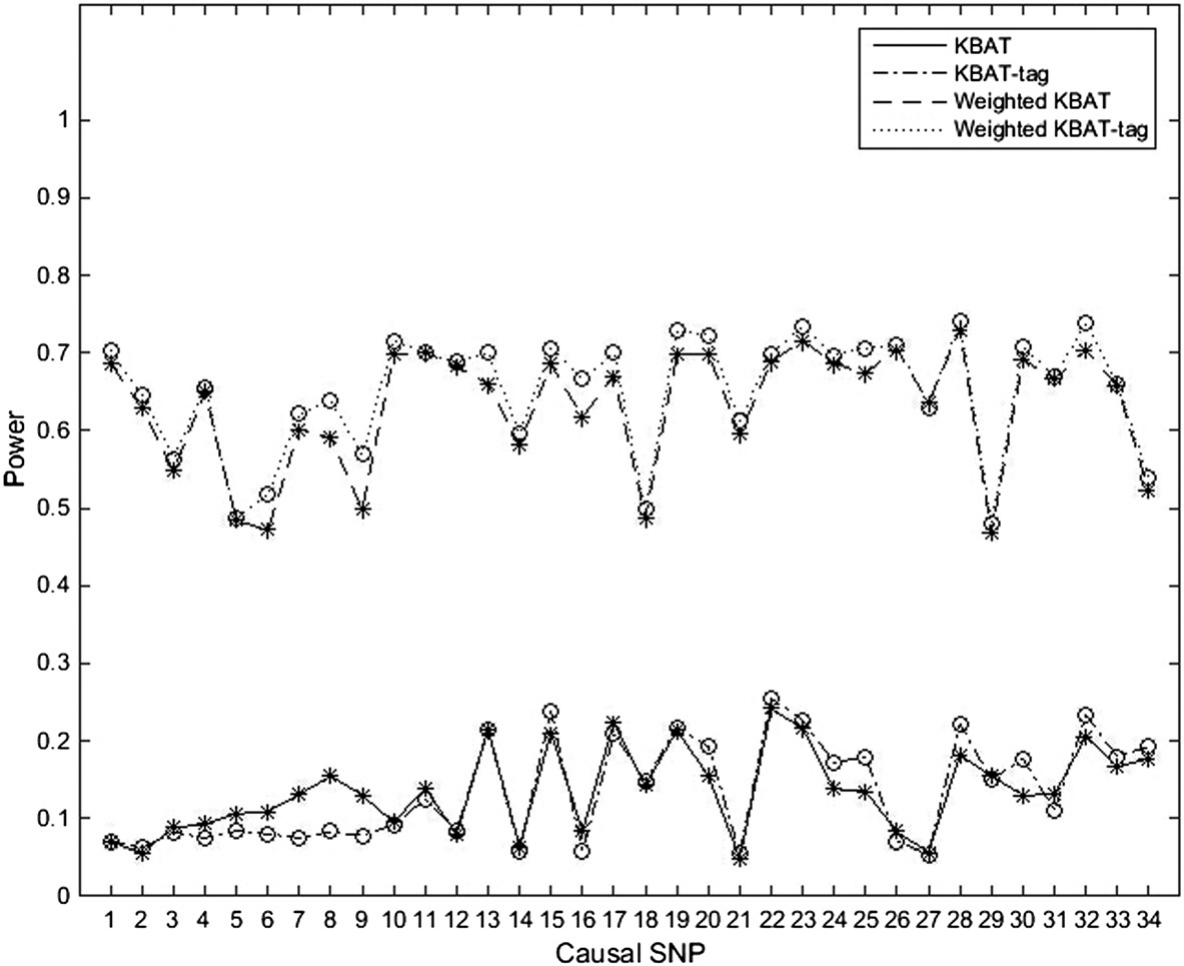
**Power comparisons of different SNP-sets for KBAT.** This shows the power comparisons of KBAT, KBAT-tag, Weighted KBAT and Weighted KBAT-tag at the significant level of 0.05.

To evaluate the powers of KBAT, KBAT-tag, weighted KBAT and weighted KBAT-tag, we simulate 1000 replicated data sets in scenarios 2–9. Figure 1 plots the powers of them in scenario 2. As a whole, the powers of the tag SNP-set-based tests on the basis of KBAT are higher than the corresponding original SNP-set-based tests. That is to say, the selected tag SNP plays an important role in increasing the power of statistical test by obtaining information from the SNPs with high LD. But when we regard the 6^th^, 7^th^, 8^th^ and 9^th^ SNP respectively as the causal SNP, the powers of tests based on tag SNP-set are evidently lower than the one based on original SNP-set of KBAT. We think the main reason is the high LD between the SNPs. Namely, the very high LD exists between multi-SNPs and the causal SNP. This makes the test power reduce due to losing too much information when forming the tag SNP-set. Obviously, each tag SNP in the tag SNP-set plays a different role in detecting disease association. Therefore we come to an idea that each SNP in the tag SNP-set is assigned a different value weighted by the *χ*^2^ statistic of this SNP. Figure 1 shows that, in the weighted case, the power of test based on tag SNP-set is better than that based on original SNP-set.

In order to further study the performance of our method under more complex simulation data sets, we conduct scenarios 3–9. Each data set has two causal SNPs designated randomly. Table 4 lists the powers of KBAT, KBAT-tag, weighted KBAT and weighted KBAT-tag in scenario 3–9. In un-weighted cases, the powers of KBAT based on tag SNP-set are higher than those based on original SNP-set except for few scenarios, while these exceptions do not arise in weighted case.

**Table 4:** **Powers of KBAT under the assumption of two causal SNPs at the significance level of 0.05**

**Scenario**	**3**	**4**	**5**	**6**	**7**	**8**	**9**
KBAT	0.099	0.067	0.287	0.264	0.1	0.128	0.3
KBAT-tag	0.111	0.06	0.348	0.297	0.105	0.114	0.241
Weighted KBAT	0.562	0.524	0.762	0.544	0.64	0.744	0.478
Weighted KBAT-tag	0.583	0.545	0.795	0.593	0.674	0.75	0.482

### The further validation using SKAT

**Figure 2:**
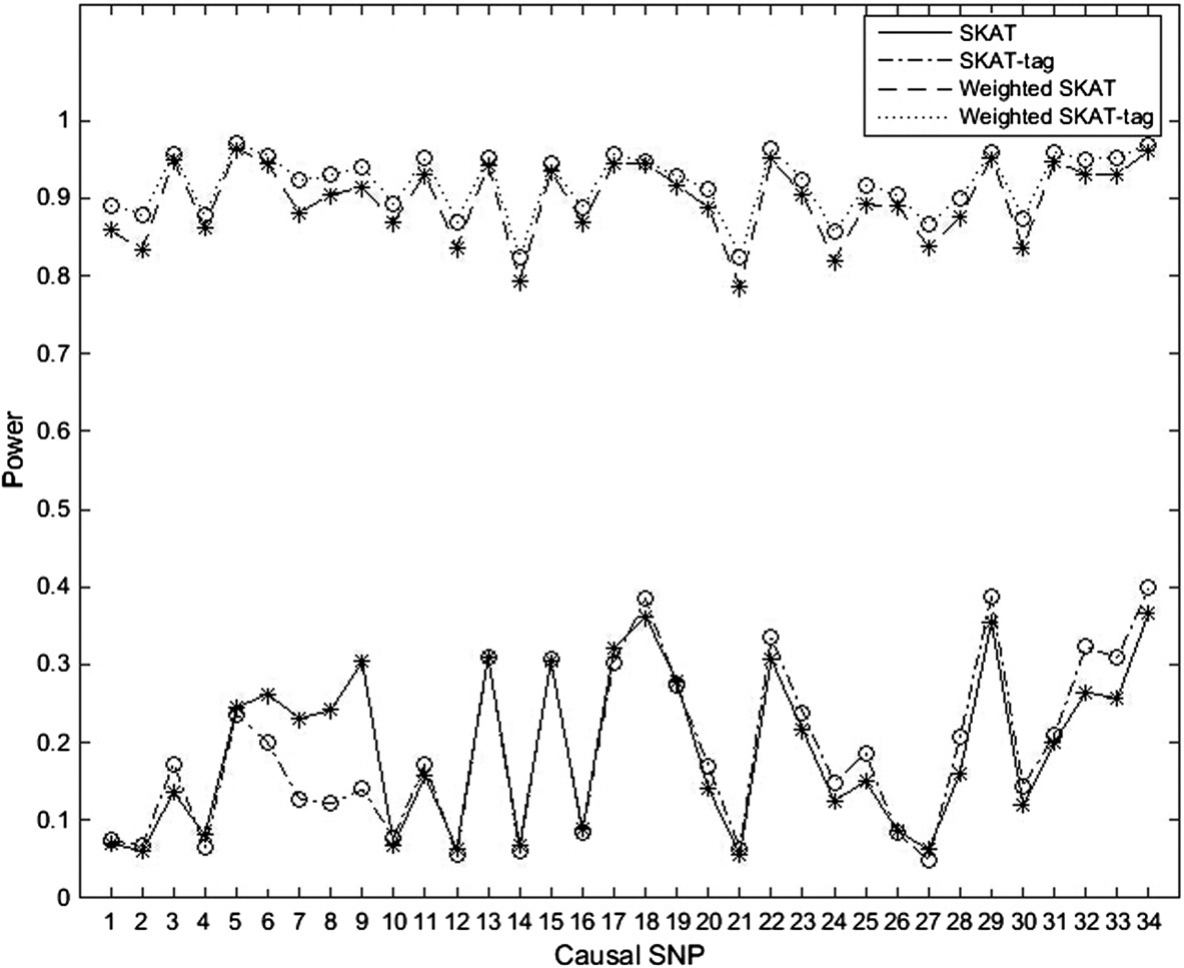
**Power comparisons of different SNP-sets for SKAT.** This shows the power comparisons of SKAT, SKAT-tag, Weighted SKAT and Weighted SKAT-tag at the significant level of 0.05.

**Figure 3:**
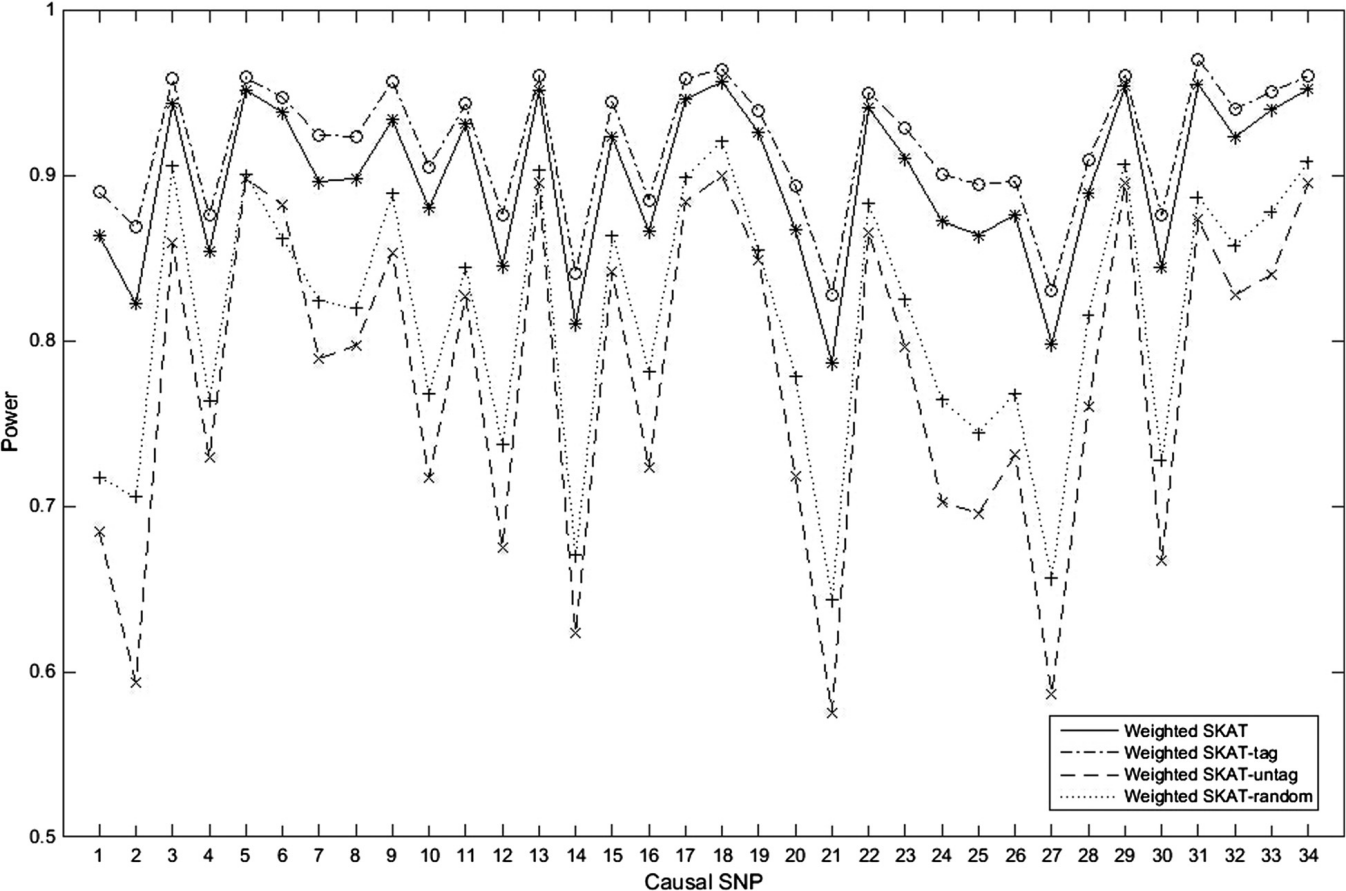
**Power comparisons of different SNP-sets for weighted SKAT.** It indicates the comparisons of the powers of the weighted SKAT based on the original SNP-set (weighted SKAT), all selected tag SNPs (weighted SKAT-tag), all remaining SNPs (weighted SKAT-untag) and a randomly selected subset (weighted SKAT-random) at the significant level of 0.05 respectively.

To further verify the performance of our method, we apply it on SKAT. Table 5 shows that the type I error of our method can be controlled. Figure 2 plots the power comparison of SKAT, SKAT-tag, Weighted SKAT and Weighted SKAT-tag in scenario 2 and Table 6 lists their powers in scenario 3–9. The results also demonstrate our proposed weighted tag SNP-set analytical method is effective in disease association. To estimate the influence of the selection of the tag SNP-set on the test power, we compare the powers of the weighted SKAT-tag based on four types of tag SNP-sets: the original SNP-set, all tag SNPs selected by our proposed algorithm of selecting, all remaining SNPs and a randomly selected subset. Figure 3 indicates that the power of the weighted SKAT-tag based on the tag SNP-set selected by our proposed algorithm is the largest.

**Table 5:** **Type I error rate in scenario 1 for SKAT**

**Significance level**	**SKAT**	**SKAT-tag**	**Weighted SKAT**	**Weighted SKAT-tag**
0.05	0.049	0.048	0.05	0.048
0.01	0.0092	0.0098	0.0104	0.0096
0.001	0.0008	0.0006	0.001	0.0012

**Table 6:** **Powers of SKAT under the assumption of two causal SNPs at the significance level of 0.05**

**Scenario**	**3**	**4**	**5**	**6**	**7**	**8**	**9**
SKAT	0.16	0.1	0.265	0.508	0.13	0.123	0.674
SKAT-tag	0.207	0.1	0.334	0.539	0.132	0.114	0.637
Weighted SKAT	0.945	0.903	0.939	0.977	0.888	0.932	0.99
Weighted SKAT-tag	0.952	0.918	0.953	0.979	0.921	0.947	0.995

## Discussion

In this research, we proposed a novel powerful method-weighted Tag SNP-set analytical method, which uses weighted tag SNP-set-based test instead of the original SNP-set-based test. We also designed a new fast algorithm of selecting tag SNPs and treated *χ*^2^ statistic of individual SNP as its weight in the study of disease association. In our method, we only need to genotype the tag SNPs instead of all SNPs in original SNP-set, which greatly reduces the cost of genotyping. To illustrate the effective of our method, we applied it to the test of SKAT and KBAT respectively and conducted intensive simulations under nine scenarios. The results indicated that weighted Tag SNP-set analytical method is an attractive alternative approach in SNP-set analysis. It is worth mentioning that we only applied our method to the test of SKAT and KBAT of qualitative traits, but, theoretically, it is also suitable for all statistical tests of qualitative traits and quantitative traits. We will verify its effective in the future study.

### Power improved

Power and Type I error are two important standards in statistical test. In our proposed weighted tag SNP-set analytical method, the power is increased greatly under the condition of protecting the type I error. We also note that regardless of the tag SNP-set, the curve patterns of the powers are very similar in Figure 3. This indicates the relative size of the power of the test is determined by the LD structure between causal SNP and other SNPs. From Table 4 and Table 6, we also find that the power has no direct relationships with that whether the causal SNP is genotyped or not and the power has positive correlation with the mean *R*^2^ between causal SNP and all genotyped SNPs. This further verifies that the LD structure between causal SNPs and other SNPs impacts the relative size of the power.

### New fast algorithm of selecting tag SNPs

Obviously, the quality of the tag SNP-set impacts the test power directly because our test is performed between the tag SNP-set and disease phenotype. In the study, we selected the tag SNP-set using the LD structure information among SNPs. Firstly we established the complex network, whose nodes are SNPs and edges are the relationships of LD between SNPs, then divided it into many subsets by a threshold, and finally selected a SNP from each subset as the tag SNP to form a new set regarded as tag SNP-set. It took less than 1 minute to select 58 tag SNPs from 169 SNPs on a server (Intel(R) Core(TM) i3-3240 T CPU @2.90GHz 2.90GHz, 4GB Windows 8). During forming the tag SNP-set, threshold *t* is an important parameter. When *t* = 1_,_ each SNP represents itself and tag SNP-set is the same as original SNP-set. If *t* = 0, only one SNP is included in tag SNP-set and the analysis is similar to Max-Single method. We tested different values of *t* in our simulations, and the comparison showed that threshold has a great influence on power and *t* = 0.9 is relatively the best to improve power.

### Reduction of the cost of genotyping

Our proposed tag-SNP-based analytical method only needs to test genotypes of tag SNP loci instead of all loci of all subjects. For example, the original SNP-set used in our simulations consists of 169 SNPs and 58 SNPs (about 1/3 of the original SNP-set) of forming the tag SNP-set are showed in Table 7 when regard rs3803189 as the causal SNP in scenario 1. That is to say, the tag SNP-set-based method saves nearly 2/3 of the cost of genotyping relative to original SNP-set-based one. This also happens in other situations and that how much can be saved relies on the LD structure of the original SNP-set and the set of threshold.

**Table 7:** **The selected tag SNPs when regard rs3803189 as a causal SNP**

**Causal SNP**	**rs3803189**
The selected tag SNPs	2 4 5 7 9 10 13 15 16 23 29 31 34 37 40 58 59 60 61 62 64 65 67 68 69 72 75 79 80 81 83 85 89 91 94 103 108 111 116 118 119 120 121 125 127 129 134 136 139 143 153 155 157 158 159 166 167 168

This is an example with 169 original SNPs and each number represents a tag SNP.

Although there are many advantages in our method, limitations also exist. We only used simulative datasets to evaluate the effectiveness of our method, and did not apply the method to the real disease data. In addition, the set of threshold *t* is difficult and it determines the size of the tag SNP-set, which further greatly impacts the test power and influences the cost of genotyping.

## Conclusions

We proposed a weighted tag SNP-set analytical method involving the selection of tag SNP-set from original SNP-set and the description of status of each tag SNP-set. Based on gene *HTR2A* and the LD structure of the CEU samples of the International HapMap Project under various model parameters, our simulation studies confirmed that the weighted tag SNP-set analytical method is efficient in SNP-set analysis of GWAS. In our simulative experiments, we also demonstrated that tag SNP-set impacts the test power greatly. So we designed a fast algorithm of selecting tag SNP-set with most of information of original SNP-set, and the power of the test based on our selected tag SNP-set is the highest in our simulations. The proposed weighted function provides a better description for the status of each tag SNP according to the comparisons between weighted cases and un-weighted cases.

## Abbreviations

GWAS: Genome-wide association study
LD: Linkage disequilibrium
SNP: Single nucleotide polymorphism
KBAT: Kernel-based association test
SKAT: Sequence kernel association test
MDMR: Multivariate distance matrix regression
AM: Allele match kernel
AS: Allele share kernel
PCA: Principal component analysis
PC: Principal component
